# P-1577. Healthcare utilization, antibiotic spectrum index, and outcomes associated with complicated urinary tract infections in the United States

**DOI:** 10.1093/ofid/ofae631.1744

**Published:** 2025-01-29

**Authors:** Sonali Advani, Helen L Zhang, Tetsu Ohnuma, Alexandria A Spellman, Karthik Raghunathan, Vijay Krishnamoorthy, Deverick J Anderson, Kenneth Schmader, Charles Scales

**Affiliations:** Duke University School of Medicine, Durham, North Carolina; Durham VA Medical Center, Durham, North Carolina; Duke University, Durham, North Carolina; Duke University Medical Center, Durham, North Carolina; Duke University, Durham, North Carolina; Duke University, Durham, North Carolina; Duke Center for Antimicrobial Stewardship and Infection Prevention, Durham, NC; Duke University School of Medicine, Durham, North Carolina; Duke University School of Medicine, Durham, North Carolina

## Abstract

**Background:**

Despite a recent increase in the burden of complicated urinary tract infections (cUTI), contemporary data on healthcare utilization, outcomes and antimicrobial use related to cUTIs are lacking. We describe healthcare utilization, inpatient antibiotic use, and mortality associated with cUTI across a nationwide US hospital network.

**Figure:**
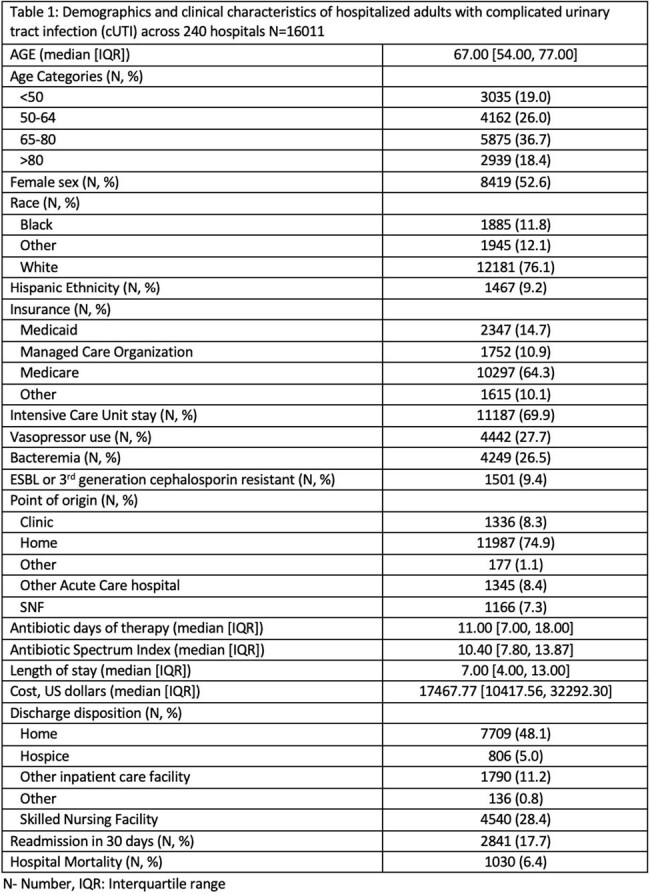

**Methods:**

This cohort study included inpatients aged ≥18 years with cUTI (using ICD-10 diagnostic codes) from 240 hospitals in the Premier Healthcare Claims Database between January 2016 – June 2020. Demographics, clinical characteristics, antibiotic use, and healthcare utilization measures were evaluated. In addition to antibiotic days of therapy, we calculated daily average Antibiotic Spectrum Index (ASI) which measures the spectrum of antimicrobial action based on number of organisms susceptible to that drug (total = 14 organism categories, e.g. ASI of penicillin is 2 while meropenem is 10, validated by Gerber et. al, PMID: 28560946). Primary outcome of inpatient mortality was assessed in a multivariable logistic regression model.

**Figure:**
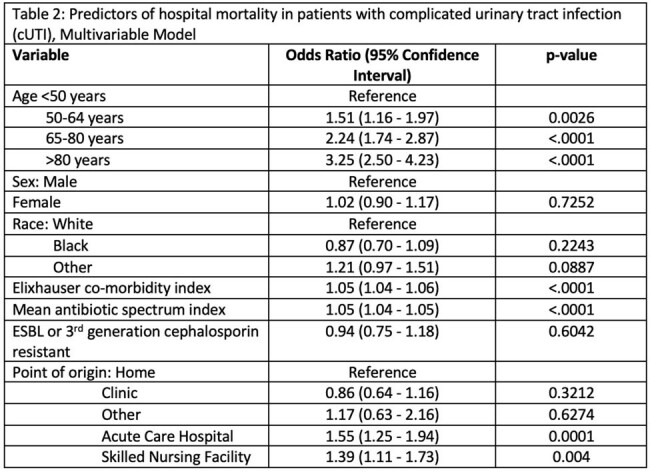

**Results:**

In this cohort of 16,011 inpatients with cUTI, median age was 67 years (IQR 54- 77], 52.6% (N=8419) were female and 76.1% (N=12181) were white (Table 1). Over two thirds of patients with cUTI required intensive care unit care, while over 25% were bacteremic and required vasopressors. Median duration of antimicrobial therapy was 11 days [IQR: 7, 18] and median daily ASI was 10.40 [IQR: 7.80, 13.87]. Inpatient mortality was 6.4% and 30 day readmission rates were 17.7%. On multivariable analysis, significant risk factors for inpatient mortality in patients with cUTI included increasing age, with highest risk over 80 years of age (adjusted odds ratio (aOR) 3.25 (2.50 - 4.23); higher Charleston’s comorbidity index (aOR 1.05 (1.04 - 1.06)); admission from skilled nursing facility (aOR 1.39 (1.11 - 1.76) and higher ASI (aOR 1.05 (1.04 - 1.05, Table 2)).

**Conclusion:**

In our cohort of 240 US hospitals, older adults and men experienced more than half of inpatient cUTIs. A large proportion of cUTIs were associated with high severity of illness requiring higher acuity of care. Older age, more comorbidities, and higher ASI were associated with increased mortality in patients with cUTI.

**Disclosures:**

**Sonali Advani, MBBS, MPH, FIDSA**, Biomerieux: Advisor/Consultant|GSK: Advisor/Consultant|Locus Biosciences: Advisor/Consultant|Sysmex America: Advisor/Consultant **Charles Scales, MD MSHS**, BMS: Grant/Research Support|Exelixis: Grant/Research Support|Flume Catheter: Grant/Research Support|Lilac Therapeutics: Advisor/Consultant|Merck: Grant/Research Support|Pfizer: Grant/Research Support

